# How PI3K-derived lipids control cell division

**DOI:** 10.3389/fcell.2015.00061

**Published:** 2015-09-30

**Authors:** Carlo C. Campa, Miriam Martini, Maria C. De Santis, Emilio Hirsch

**Affiliations:** Department of Molecular Biotechnology and Health Sciences, University of TurinTorino, Italy

**Keywords:** phosphoinositides, GTPases, cytoskeleton dynamics, PI3 kinase, trafficking, cell division

## Abstract

To succeed in cell division, intense cytoskeletal and membrane remodeling are required to allow accurate chromosome segregation and cytoplasm partitioning. Spatial restriction of the actin dynamics and vesicle trafficking define the cell symmetry and equivalent membrane scission events, respectively. Protein complexes coordinating mitosis are recruited to membrane microdomains characterized by the presence of the phosphatidylinositol lipid members (PtdIns), like PtdIns(3,4,5)*P*_3_,PtdIns(4,5)*P*_2_, and PtdIns(3)*P*. These PtdIns represent a minor component of cell membranes, defining membrane domain identity, ultimately controlling cytoskeleton and membrane dynamics during mitosis. The coordinated presence of PtdIns(3,4,5)*P*_3_ at the cell poles and PtdIns(4,5)*P*_2_ at the cleavage furrow controls the polarity of the actin cytoskeleton leading to symmetrical cell division. In the endosomal compartment, the trafficking of PtdIns(3)*P* positive vesicles allows the recruitment of the protein machinery required for the abscission.

## Introduction

Although they represent < 1% of total cellular lipids, PtdIns (PtdIns) are important spatial and temporal regulators of cellular signaling located on the cytoplasmic leaflet of cellular membranes. Historically, PtdIns have been related with cell proliferation, migration, and vesicular trafficking while more recently they have been associated with metaphase progression, spindle orientation and cytokinesis control (Echard, 2012). The myoinositol headgroup of PtdIns can be reversibly phosphorylated/dephosphorylated by numerous selective cytosolic enzymes. Among them, the phosphatidylinositol 3-kinases (PI3Ks) represent the most common family of lipid kinases that generate PtdIns(3)*P*, PtdIns(3,4)*P*_2_, and PtdIns(3,4,5)*P*_3_. PI3Ks are enzymes that play a key role in the regulation of signal transduction, cell metabolism and survival. Based on their primary structure, mechanism of action and specific products, PI3K are subdivided in three classes. PI3Kα, PI3Kβ, PI3Kδ, and PI3Kγ belong to class I, PI3K-C2α, PI3K-C2β, and PI3K-C2γ to class II, and vacuolar protein sorting 34 (Vps34) to class III (Martini et al., 2014).

Class I PI3Ks family is composed by four different p110 isoforms (α, β, γ, and δ) and the related regulatory subunits. For p110α, p110β, and p110δ, the most common regulatory subunit is the p85 protein (PIK3R1), while p110γ associates with the p101 and p84/p87 regulatory subunits. Class I PI3K occurs as obligatory dimers in cells (Geering et al., 2007) and can transduce signals received from tyrosine kinase receptors (RTKs), G protein-coupled receptors (GPCRs) and active RAS. Although the majority of efforts have so far focused on class I PI3K isoforms, increasing evidence is pointing to the importance of class II enzymes in cell proliferation and survival (Franco et al., 2014; Braccini et al., 2015). Class II enzymes are large molecules ranging from 166 to 190 kDa with the PI3K catalytic core flanked by extended N- and C-terminal arms (Falasca and Maffucci, 2007). Differently from other PI3Ks that function as heterodimers, they do not associate to an adaptor protein but their N- and C-termini contain multiple protein and lipid interaction domains that favor protein-protein and protein-lipid interactions (Campa et al., 2015b). The precise nature of the class II PI3K substrates and lipid products is still debated. Unlike class I enzymes, that only produce PtdIns(3,4,5)*P*_3_ *in vivo*, class II members are thought to act similarly to class III and mainly generate PtdIns(3)*P in vitro* and *in vivo* (Falasca and Maffucci, 2007). The class II PtdIns(3)*P* derived-production accounts for the 20% of the total PtdIns(3)*P* present in the cells and controls critical processes like autophagy and endocytic recycling (Vanhaesebroeck et al., 2010; Franco et al., 2014, 2015). Nonetheless, PI3K-C2α and PI3K-C2γ can also produce PtdIns(3,4)*P*_2_ (Vanhaesebroeck et al., 2010; Braccini et al., 2015) and PI3K-C2α has been also reported to produce PtdIns(3,4,5)*P*_3_ in selective subcellular locations (Gaidarov et al., 2001). The class III PI3K, Vps34, was first identified as a component of the vacuolar sorting system in *Saccharomyces cerevisiae* (Backer, 2008). The homolog in mammalian cells, PI3KC3, and its adaptor subunit, p150/Vps15, play an important role in the context of autophagy, endosomal trafficking, nutrients sensing and cytokinesis through the generation of PtdIns(3)*P* (Bader et al., 2005).

PIs production is tightly regulated not only by several kinases but also by specific phosphatases that allow the correct lipid turnover. In particular, the termination of signaling by degradation of PtdIns(3,4,5)*P*_3_ is known to be mediated by multiple inositol polyphosphate phosphatases. Several studies have identified three major PtdIns(3,4)*P*_2_/PtdIns(3,4,5)*P*_3_-degrading enzymes: (a) phosphatase and tensin homolog (PTEN), that dephosphorylates the 3-position of PtdIns(3,4,5)*P*_3_ to produce PtdIns(4,5)*P*_2_ (Di Cristofano and Pandolfi, 2000), (b) Src-holomology 2 (SH2)-containing inositol 5′-phosphatase (SHIP), which dephosphorylates PtdIns(3,4,5)*P*_3_ into PtdIns(3,4)*P*_2_ (Damen et al., 1996) and inositol polyphosphate 4-phosphatase (INPP4), which hydrolyzes the 4-position of PtdIns(3,4)*P*_2_ (Gewinner et al., 2009). Two INPP4 phosphatases have been described, INPP4A and INPP4B, with specific expression patterns and subcellular localization, that interact with the plasma membrane via PtdIns interactions (Shearn and Norris, 2007). PTEN is the most characterized PIs phosphatase with a lipid-binding domain that mediates its anchorage to the plasma membrane. The major role of PTEN is to buffer PtdIns(3,4,5)*P*_3_ levels thus inhibiting PI3K signaling and contributing to regulate cell growth, survival, motility and metabolism. The localization of the enzymes that interconvert PtdIns is tightly regulated in space and time in order to specifically distribute PtdIns across the cellular membranes (Pitcher et al., 1998; Heo et al., 2006; Lemmon, 2008; Newton, 2009; Kutateladze, 2010; Echard, 2012).

## Mechanism of phospholipid recognition

PtdIns are signaling platforms that allow the recruitment of proteins in precise subcellular locations. This is achieved by specific PtdIns-binding proteins that constantly sample the membrane environment, moving toward PtdIns-enriched sites. However, during mitosis, these protein complexes are controlled both at temporal and spatial level by several mechanisms that mediate phospholipid recognition and biological effects.

The understanding of the biological role of PtdIns originates from the observation that phospholipase C delta 1 (PLC-δ1) binds with high affinity to PtdIns(4,5)*P*_2_ through its PH domain that allows the recruitment of the protein at the cell membrane after cell stimulation (Garcia et al., 1995; Lemmon et al., 1995; Várnai et al., 2002). Afterwards, the isolation of several PtdIns binding domain selective for different PtdIns species, such as FERM (band Four point one, Ezrin, Radixin, and Moesin) (Chishti et al., 1998), FYVE (Fab-1, YGL023, Vps27, EEA1) (Gaullier et al., 1998), PX (Phox) (Gaullier et al., 1998; Cheever et al., 2001), ENTH/CALM (Epsin N-terminal homology domain/Clathrin Assembly Lymphoid Myeloid) (Ford et al., 2001, 2002) and PROPPIN (β-propellers) (Michell et al., 2006) points out the importance of phospholipid recognition as a mechanism to protein localization and/or activation in specific subcellular membrane compartments (Kutateladze, 2010).

From a biochemical point of view, this mechanism is based on: (i) the lipid headgroup size, (ii) the electrostatic charge of the headgroup, (iii) the lipid packing properties. In PtdIns, the presence of one or more phosphate headgroups makes these lipids larger than other phospholipids, such as phosphatidylcholine (PC) (Zhou et al., 1997; Bradshaw et al., 1999). This larger size allows the protrusion of the head group in the aqueous phase, making this lipid functional for biological processes (McLaughlin et al., 2002). In addition, the phosphate groups confer a negative charge to the lipid headgroup, promoting the engagement in electrostatic interactions with protein containing polybasic domains (Yeung et al., 2006; Magalhaes and Glogauer, 2010).

Finally, the ratio between polar heads and acyl chain determines the lipid packing properties (van Den Brink-van Der Laan et al., 2004; Janmey and Kinnunen, 2006) that, in combination with the headgroup charge, favor the generation of curved membrane region recognized by specific protein domain, like member of Bin–Amphiphysin–Rvs (BAR) domain protein family (Peter et al., 2004; Vanni et al., 2014). These features highlight a critical concept of PtdIns biological function, i.e., the spatio-temporal protein recruitment to the cellular membrane by protein-lipid interaction. In this context, several mechanisms have been identified to control protein recruitment to subcellular compartment based on PtdIns-mediated recognition, such as: (i) high affinity (ii) coincidence detection (synergistic interaction of protein and/or lipids to increase protein-PtdIns binding affinity) mediated by protein-protein and protein-lipid interaction and (iii) PtdIns-mediated conformational change. The major fraction of PtdIns-binding proteins recognizes PtdIns with low affinity, indicating that membrane recognition by protein domain cannot act as a principal mechanism for membrane recruitment (Kavran et al., 1998; Lemmon, 2007). Accordingly, isolated Dynamin PH domain is unable to be localized in membrane (Kavran et al., 1998; Klein et al., 1998). Nonetheless, the dimerization of multiple dynamin PH domain significantly enhances the affinity for PtdIns-containing lipid membrane from mM to pM affinity (Kavran et al., 1998). In addition, the PtdIns can mediate a conformational change that allows protein activation, independently from effects on membrane targeting. In conclusion, the targeting and activation of cytosolic protein to intracellular membrane define the timing of protein function during biological process. These mechanisms are important during the cell cycle in order to define precise temporal initiation, duration, and termination of mitotic events.

In the following sections, we will describe the involvement of PI3K-derived lipids and their interactors during mitosis, focusing on mechanism at the base of mitotic spindle orientation and promoting cell abscission.

## Functional interaction of PI3K with Cdk promotes entry to mitosis

**Figure 1:**
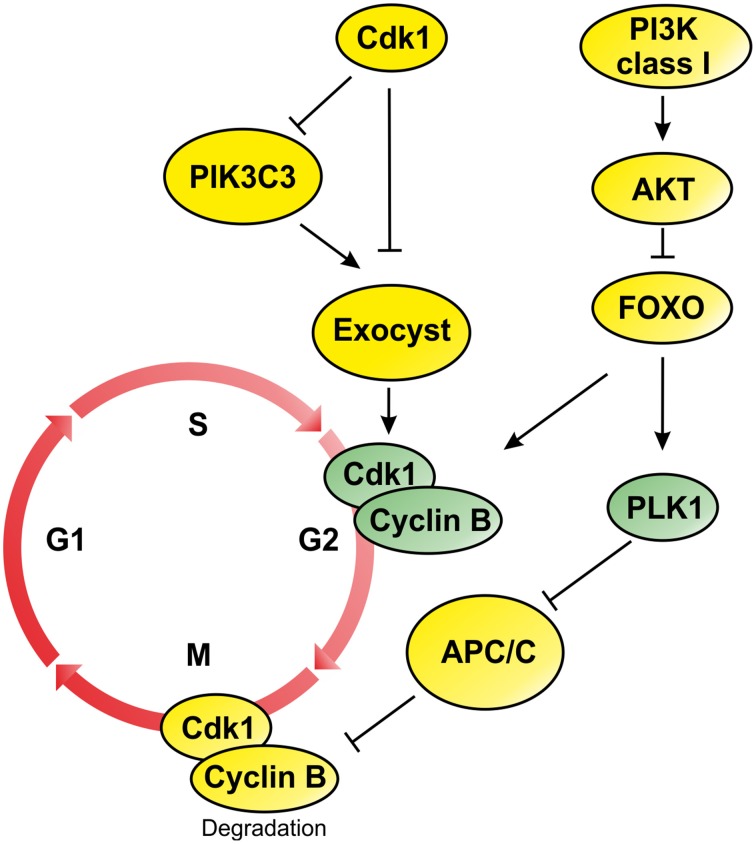
**Schematic representation of PI3Ks signaling during G2/M progression and mitosis phase**. During mitosis attenuation of PI3K/AKT pathway drives activation of FOXO proteins and transcription of genes involved in G2/M transition such as CdK1, Cyclin B, and PLK1. On the contrary, the inactivation of PI3KC3 is regulated by Cdk1 that inactivates components of the Exocyst involved in the mitotic progression. In addition, inhibition of Exocyst affects Cyclin B transcription promoting mitotic progression (Post-translational modification are represented by yellow circles, while elements controlled by transcriptional regulation are colored in green).

Several studies have demonstrated that lipids produced by the PI3K protein family are key regulator of cell cycle progression during the G1/S transition. Acting downstream receptors tyrosine kinase (RTK) and G protein–coupled receptors (GPCR), PI3Ks direct the initiation of DNA synthesis through the activation of several signaling transduction events mainly mediated by the RAC-alpha serine/threonine kinase (AKT) (Figure 1). In basal conditions, AKT is maintained in its inactive form by the interaction of its PH and kinase domains (“PH-in conformation”), that prevents PDK1-mediated AKT-Thr308 phosphorylation. On the other hand, AKT-PH domain interaction with PtdIns and the consequent conformational change (“PH-out”) promote the phosphorylation of residue Thr308 by PDK1 (Calleja et al., 2007). Activation of AKT induces the phosphorylation of several downstream effectors, including the family of Forkhead (FKH) transcription factors (TF) FoxO (Figure 1). During G2 phase, attenuation of PI3K/AKT signaling regulates the translocation from cytosol to the nucleus of FoxO proteins that, in turn, regulate the expression of several important mitotic regulators, such as Cyclin B and Polo-like-kinase (PLK1) (Figure 1) (Alvarez et al., 2001; Laoukili et al., 2005). Following Cyclin B1 phosphorylation by PLK1, the cyclin B1/Cdk1 complex is targeted to the nucleus during prophase (Toyoshima-Morimoto et al., 2001; Jackman et al., 2003). This process is controlled by β1-Integrin activation that triggers PI3K function during M phase (Xu et al., 2009) and regulates the orientation of mitotic spindle.

Moreover, attenuation of PI3K signaling was also observed for PIK3C3 the class III member of PI3K family (Furuya et al., 2010). During mitosis, PI3KC3 is phosphorylated by Cdk1, which negatively regulates its interaction with Beclin 1 during mitosis (Figure 1) (Furuya et al., 2010). Moreover, Cdk1 also phosphorylates the component of the exocyst EXO84 (Figure 1), a PIK3C3 binding partner, which is required for docking and fusion of secretory vesicles at the site of membrane growth, affecting exocytosis and cell surface expansion (Bodemann et al., 2011; Luo et al., 2013). The coordination between membrane trafficking and cell cycle progression provides a molecular mechanism by which cell size is controlled during the cell cycle (Luo et al., 2013). However, loss of function of Sec6, another component of the exocysts, results in decreased cell size leading to low level of activation of Cdk1 in early mitosis and failure to activate Cyclin B2 transcription (Figure 1) (TerBush et al., 1996; Anastasia et al., 2012). These studies suggest that the entry in the mitotic phase depends on cell size regulation that is controlled by the activation of PI3K/AKT signaling axis and by the inhibition of PI3KC3 function by Cdk1.

## Cooperation of small GTPases and PI3Ks contributes to metaphase progression and spindle orientation

Mitotic spindle orientation determines the axis of cell division during prophase and allows proper chromosome segregation through metaphase/anaphase transition and it depends on interactions of astral microtubules with the cell cortex (Siller and Doe, 2009). This interaction is mediated by a microtubule plus-end-tracking protein family (+Tips), which is composed by several members, including dynein/dynactin complexes (Toyoshima et al., 2007). Dynein/dynactin is a minus-end microtubule (MT) motor protein complex that localizes to the cell cortex in metaphase, where it regulates spindle orientation. In particular, during metaphase, dynactin, a dynein-binding partner, is accumulated at the midcortex, the midsection of the cell cortex in a PtdIns(3,4,5)*P*_3_-dependent manner (Toyoshima et al., 2007). Lack of class I PI3K enzymes results in the mislocalization of the dynein-associated dynactin (Toyoshima et al., 2007). This alteration is rescued by exogenous addition of PtdIns(3,4,5)*P*_3_ but not PtdIns(3,4)*P*_2_ or PtdIns(4,5)*P*_2_, suggesting an involvement of PtdIns(3,4,5)*P*_3_ in mitotic spindle orientation (Toyoshima et al., 2007). Thus, PI3Ks regulate the recruitment of dynein/dynactin complex at the midcortex in a PtdIns(3,4,5)*P*_3_-dependent manner. To permit correct localization of PtdIns(3,4,5)*P*_3_ at the mitotic cortex, the class I PI3K activity is counterbalanced with PTEN, that allows spatial restriction of PtdIns(3,4,5)*P*_3_ (Toyoshima et al., 2007). Consistently, depletion of PTEN causes expansion of the PtdIns(3,4,5)*P*_3_ domain over the whole cortex and is associated with dynactin mislocalization and spindle orientation defects (Toyoshima et al., 2007).

The production of PtdIns(3,4,5)*P*_3_ and consequently the localization of dynactin at the cell cortex is controlled by the activity of Cdc42, a member of the Rho GTPase family, involved in the modulation of actin and microtubules dynamics (Etienne-Manneville and Hall, 2002). In particular, the junctional adhesion molecule-A (JAM-A) activates Cdc42, that stimulates PI3K activation and PtdIns(3,4,5)*P*_3_ accumulation at the midcortex, regulating the formation of the cortical actin cytoskeleton and the cortical localization of dynein during mitosis (Tuncay et al., 2015). During the M phase, knock-down of Cdc42 suppresses PI3K activity and consequently decreases PtdIns(3,4,5)*P*_3_ levels, disrupting localization of the dynein/dynactin complex and inducing spindle misorientation (Mitsushima et al., 2009). This mechanism has been suggested to be promoted by a positive feedback that stimulates Cdc42 activation through PtdIns(3,4,5)*P*_3_ production (Mitsushima et al., 2009). Accordingly, Cdc42 activation is controlled by PI3K enzymes (Yang et al., 2012). As a consequence, exogenous addition of PtdIns(3,4,5)*P*_3_ in silenced Cdc42 cells reduces PI3K activity during the M phase (Toyoshima et al., 2007; Mitsushima et al., 2009). Therefore, PI3K-mediated production of PtdIns(3,4,5)*P*_3_ establishes a positive feedback loop mediated by Cdc42, which controls dynein recruitment at the midcortex, proper spindle orientation and metaphase progression.

In addition to the key role of PI3K/PTEN activity in controlling mitotic spindle and cell polarity (Toyoshima et al., 2007), PTEN has been identified to maintain mitotic checkpoint (Gupta et al., 2009) and to control mitotic progression (Choi et al., 2014). The authors reported that Ser-380 phosphorylation by PLK1 is associated with the accumulation of PTEN on chromatin and promotes its mitotic function. Expression of phospho-deficient mutant, but not wild-type PTEN, caused enhanced mitotic exit, suggesting that Ser-380 phosphorylation may play a role in stabilizing PTEN during mitosis.

These findings show that PI3Ks together with PTEN phosphatase have a significant role in regulating mitotic progression. Several studies suggest that nuclear/chromatin PTEN modulates the activity of genes that mediate homologous recombination (Choi et al., 2013, 2014). Since PTEN controls chromosomal stability through its physical association with centromere protein in a phosphatase-independent manner, it has been proposed as a major guardian of the maintenance of genomic stability (Shen et al., 2007).

## Attenuation of PI3K signaling during anaphase and telophase stages

Anaphase and telophase are technically the last phases of mitosis, during which the duplicated centromeres separate and the sister chromatids reach opposite poles. Previous reports indicate that the efficient execution of the mitotic program requires an attenuation of PI3K/AKT pathway during the late mitotic stages so that Forkhead transcription factors (FKH-TF) can induce the synthesis of important mitotic proteins, like PLK1 (Figure 1). In fact, the expression of constitutively active PI3K or AKT causes cells to arrest in telophase, with high levels of Cyclin B protein (Alvarez et al., 2001). Recently, Kasahara et al showed that Plk1-Ser99 is a novel mitosis-specific phosphorylation site that creates a docking site for 14-3-3γ, stimulating its catalytic activity (Kasahara et al., 2013). PLK1 Ser99-phospho-blocking mutant leads to prometaphase/metaphase-like arrest due to the activation of the Spindle Assembly Checkpoint (SAC). The selective inhibition of PI3K/AKT pathway significantly reduces the level of PLK1-Ser99 phosphorylation and delays metaphase to anaphase transition.

In conclusion, these observations demonstrate the functional importance of PI3K signaling reduction to allow proper mitotic progression. Future studies are required to deeply characterize the role of PtdIns signaling and address how PI3K/Akt pathway is inhibited during late stages of the mitosis phase.

## Spatial restraints of PtdIns drive membrane trafficking and actin remodeling during cytokinesis

**Figure 2:**
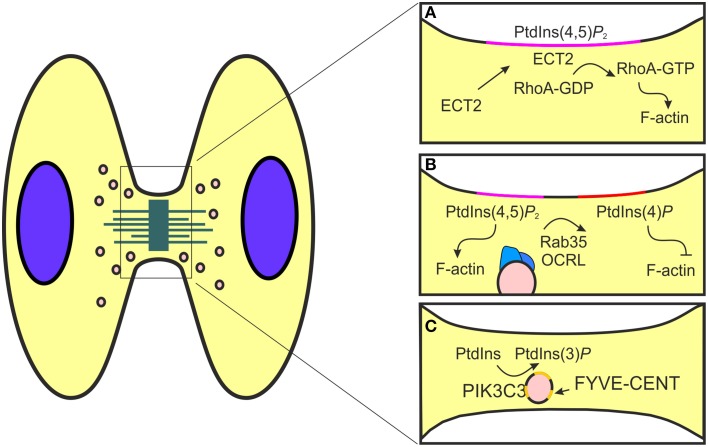
**Schematic representation of phosphorylated phosphoinositides function during cytokinesis. (A)** The formation of the mitotic furrow is controlled by the activity of RhoA, a modulator of actin polymerization. This event is promoted by PtdIns(4,5)*P*_2_ (pink) that allows the localization of the RhoA GEF ECT2 at the cell cortex. **(B)** During the post-furrowing stages, when the actin filaments need to be disassembled, components of the endocytic recycling pathways, like Rab35 (blue), are redirected to the mitotic furrow. Here, Rab35 transports OCRL (blue) that catalyzes PtdIns(4,5)*P*_2_ (pink) conversion to PtdIns(4)*P* (red) and consequently promotes actin destabilization. **(C)** PIK3C3 produces PtdIns(3)P (yellow) to control the recruitment of FYVE-CENT, a regulator of membrane abscission during cytokinesis.

Cytokinesis is the final stage of mitosis that results in the partitioning of the contents of a single cell into two (Pollard and Wu, 2010; Fededa and Gerlich, 2012). In eukaryotic cells, cytokinesis begins with the identification of the mitotic cleavage plane, which grows out from sister centrosomes in an equidistant position between the two microtubules arrays (Dogterom et al., 2005; Grill and Hyman, 2005). Next, the cleavage furrow, an actomyosin ring localized at the cleavage plane, is generated and stabilized by the midbody, a membrane stalk connecting the two daughter cells (Bringmann and Hyman, 2005; Oliferenko et al., 2009). Finally, both the actin machinery and the vesicular trafficking are involved during the abscission step through the midbody cleavage by the endosomal sorting complex (ESCRT) (Schiel and Prekeris, 2013; Chircop, 2014). After anaphase onset, there is a progressive PTEN delocalization from the polar cortex to the equator (Roubinet et al., 2011), while during cytokinesis, PTEN accumulates at the septum of dividing cells (Mitra et al., 2004) as well as at the cleavage furrow (Janetopoulos et al., 2005). Accordingly, depletion of PTEN leads to a significant enrichment of PI(3,4,5)*P*_3_ at the cortex, especially at the cleavage furrow, showing that PTEN dephosphorylates PI(3,4,5)*P*_3_ to spatially control PI(4,5)*P*_2_ levels at the mitotic cortex. In addition, OCRL enzyme is an inositol phosphatase that hydrolyses the 5-position phosphate from the inositol ring of PtdIns(3,4,5)*P*_3_, PtdIns(3,5)*P*_2_, and PtdIns(4,5)*P*_2_ (Schmid et al., 2004). OCRL is proposed to be essential for endocytic recycling, ciliogenesis and cytokinesis, and localizes mainly in the endocytic network including early endosomes and endocytic clathrin-coated pits (Erdmann et al., 2007; Ben El Kadhi et al., 2011; Dambournet et al., 2011; Conduit et al., 2012). The spatial localization of PtdIns(3)*P* and PtdIns(4,5)*P*_2_ promotes cytokinesis by two independent mechanisms involving the midbody proteins localization and the actin filaments destabilization at the cleavage furrow (Figure 2). PtdIns(3)*P* is a known regulator of autophagy, endosomal vesicle trafficking and cell signaling, enriched in endosomal membrane (Raiborg et al., 2013; Schink et al., 2013). During cytokinesis, PtdIns(3)*P* positive endosomes localize at the midbody and the inhibition of PtdIns(3)*P* synthesis by PI3K inhibitors induces cleavage furrow regression and blocks cytokinesis (Janetopoulos et al., 2005; Nezis et al., 2010). This process is controlled by class III PI3K and the depletion of PIK3C3 decreases the amount of PI(3)*P*-positive endosomes at the midbody, increasing the percentage of multinucleate cells and delaying cytokinesis completion (Sagona et al., 2010). At the midbody, PtdIns(3)*P* controls the recruitment of the PtdIns effector, Zinc finger FYVE domain-containing protein 26 (FYVE-CENT) and its binding partner tetratricopeptide repeat protein 19 (TTC19) (Figure 2) (Nezis et al., 2010). In this site, the interaction between TTC19 and its effector chromatin-modifying protein/charged multivesicular body protein 4B (CHMP4B) participates in the formation of the ESCRT-III complex, the membrane constricting protein complex responsible for the final step of abscission (Nezis et al., 2010). Depletion of FYVE-CENT or TTC19 causes cytokinesis arrest and increases the number of binucleate and multinucleate cells, in a similar manner to the depletion of PI3K-C3 or Beclin, the regulator subunits of class III PI3K (Nezis et al., 2010). In addition to PI3K-C3, also the depletion of PI3K-C2A decreases the amount of PI(3)*P*-positive endosomes and reduces endocytic recycling of protein to the primary cilium (Franco et al., 2014). PI3K-C2A exerts this function through the activation of the small GTPase Rab11 that actively controls endocytic recycling (Franco et al., 2014). During cytokinesis the activation of Rab11 promotes the interaction with members of Rab11 interacting proteins family (Rab11-FIP), like FIP3 (Eathiraj et al., 2006; Horgan and McCaffrey, 2009) that binds TSG101 (Tumor susceptibility gene 101 protein), an ESCRT-I component (Horgan et al., 2012). This interaction localizes TSG101 to midbody and promotes the completion of cytokinesis. While, depletion of Rab11 FIP3 disrupts abscission (Wilson et al., 2005).

This polarized vesicle transport mechanism is also used by PtdIns and actin regulators to promote the destabilization of the actomyosin ring, a final event required for proper abscission (Chen et al., 2012). The generation of a stable actin/myosin–based contractile ring is promoted by the activity of PI3K and PTEN enzymes (Janetopoulos et al., 2005). The coordinated localization of these enzymes allows the accumulation of PtdIns(4,5)*P*_2_, a known modulator of actin polymerization, at the cleavage furrow (Janetopoulos et al., 2005). In this site, the production of F-actin is controlled by RhoA, a member of the Rho family small GTPase activated by the RhoA guanine nucleotide exchange factor (GEF) epithelial cell transforming 2 (ECT2) (Field et al., 2005; Yüce et al., 2005; Chircop, 2014). At the steady state, ECT2 locates in the nucleus while during cytokinesis the presence of a PH domain redirect the protein to the cleavage furrow (Chalamalasetty et al., 2006; Matthews et al., 2012). The shuttling of ECT2 is promoted by the interaction between the ECT2 PH domain and phosphoinositides (Su et al., 2011; Frenette et al., 2012) (Figure 2). As a consequence, truncation of the PH domain in ECT2 impairs protein localization at the cortex (Chalamalasetty et al., 2006). Usually the interaction between GEF proteins and phosphoinositides contributes to increase the nucleotide exchange rate on the small GTPase (Rossman et al., 2005; Campa et al., 2015a). Nonetheless, the potential involvement of phosphoinositide in the control of the guanine nucleotide exchange of ECT2 remains undressed.

The function of PtdIns(4,5)*P*_2_ during cytokinesis is not limited to the promotion of furrow ingression, but also in post-furrowing stages, when the actin filaments need to be disassembled before abscission. This process requires the recruitment of proteins that convert PtdIns(4,5)*P*_2_ to PtdIns(4)*P*_2_, such as OCRL (Dambournet et al., 2011). During interphase, OCRL is localized in endocytic clathrin coated pits but it is redirected to the cleavage furrow during cytokinesis (Figure 2) (Dambournet et al., 2011; Nández et al., 2014). The localization of OCRL is controlled both by a coincidence interaction with PtdIns(4,5)*P*_2_ through its PH domain and also by elements of the vesicular trafficking machinery, like clathrin and Rab35 (Mao et al., 2009; Hou et al., 2011). Rab35 is a modulator of fast recycling pathway localized to the plasma membrane, clathrin coated vesicles and endosomes (Kouranti et al., 2006; Dambournet et al., 2011). During cytokinesis Rab35, like other component of the endocytic recycling pathway as Rab11, promotes the transport of proteins and lipids at the intercellular bridge. Whereas, Rab11-Fip3 positive endosomes promotes actin destabilization inhibiting the RhoA activity through the delivery of the p50RhoGAP, Rab35 controls OCRL localization and consequently actin disassembly directing PtdIns conversion at the cleavage furrow (Figure 2). Therefore, depletion of Rab35 or OCRL results in stable intercellular bridges due to increased amount of cortical actin and PtdIns(4,5)*P*_2_, leading to abscission failure (Ben El Kadhi et al., 2011; Dambournet et al., 2011). In this manner two independent endocytic routes controlled by Rab11 and Rab35 are involved in the regulation of actin dynamics during cytokinesis (Kouranti et al., 2006). In conclusion, PI3K and PI3K-derived PtdIns promote the recruitment of proteins at the intracellular bridge where they mediate the final scission through the destabilization of the furrow and by the localization of the ESCRT complex.

## Conclusion

It is now well established that PtdIns are key regulators of signal transduction, membrane trafficking and cytoskeleton dynamics. During the cell cycle, the localization of PtdIns is tightly regulated in space and time through the concurrent activity of several lipid kinases and phosphatases. PI3Ks represent the most common family of lipid kinases that generate PtdIns(3)*P*, PtdIns(3,4)*P*_2_, and PtdIns(3,4,5)*P*_3_. Several studies showed that increased activity of PI3K/AKT axis promotes G1/S cell cycle progression through inactivation of GSK3-beta, leading to increased cyclin D1 and inhibition of FKH-TF. At the onset of mitosis, the attenuation of PI3K pathway is required to allow transcriptional activation of FKH-TF and subsequent expression of key mitotic genes, such as Cyclin B and PLK1. On the other hand, PTEN, which counteracts the PI3Ks function, plays an important role in regulating mitotic timing and controlling chromosome integrity. In addition, class I and class III PI3Ks and related phosphatases contribute to the specific localization of protein complexes at the spindle pole during metaphase and at the intracellular bridge during cytokinesis. Despite this wealth of information, several aspects of PI3K signaling in mitosis are only now starting to emerge and future studies are likely to define new roles of PI3K signaling, better defining, for example, their function in membrane and vesicular dynamics as well as chromosomal congression and genetic stability.

### Conflict of interest statement

Carlo C. Campa and Emilio Hirsch have a pending patent related to Rab11 activity kit [102014902248060 (TO2014A000264)]. Emilio Hirsch is co-founder of Kither Biotech, a company involved in the development of PI3K inhibitors. The other authors declare that the research was conducted in the absence of any commercial or financial relationships that could be construed as a potential conflict of interest.
